# NRF2 promotes breast cancer cell proliferation and metastasis by increasing RhoA/ROCK pathway signal transduction

**DOI:** 10.18632/oncotarget.12435

**Published:** 2016-10-04

**Authors:** Chao Zhang, Hui-Jie Wang, Qi-Chao Bao, Lei Wang, Tian-Kun Guo, Wei-Lin Chen, Li-Li Xu, Hai-Shan Zhou, Jin-Lei Bian, Ying-Rui Yang, Hao-Peng Sun, Xiao-Li Xu, Qi-Dong You

**Affiliations:** ^1^ Jiangsu Key Laboratory of Drug Design and Optimization, China Pharmaceutical University, Nanjing, 210009, China; ^2^ State Key Laboratory of Natural Medicines, China Pharmaceutical University, Nanjing, 210009, China; ^3^ Department of Medicinal Chemistry, School of Pharmacy, China Pharmaceutical University, Nanjing, 210009, China

**Keywords:** NRF2, breast cancer, RhoA/ROCK pathway, cell proliferation, metastasis

## Abstract

Nuclear factor erythroid 2-related factor (NRF2) is an important transcription factor in oxidative stress regulation. Overexpression of NRF2 is associated with human breast carcinogenesis, and increased NRF2 mRNA levels predict poor patient outcome for breast cancer. However, the mechanisms linking gain of NRF2 expression and poor prognosis in breast cancer are still unclear. Here, we provide evidence that NRF2 deletion inhibits proliferation and metastasis of breast cancer cells by down-regulating RhoA. Restoration of RhoA in MCF7 and MDA-MB-231 cells induced NRF2 knockdown-suppressed cell growth and metastasis *in vitro*, and NRF2 silencing suppressed stress fiber and focal adhesion formation leading to decreased cell migration and invasion. Mechanistic studies showed that NRF2 binds to the promoter region of estrogen-related receptor α (ERR1) and may function as a silencer. This may enhance RhoA protein stability and lead to RhoA overexpression in breast cancer cell. Our findings indicate that NRF2 silencing-mediated reduction of RhoA expression contributes, at least in part, to the poor outcome of breast cancer patients with high NRF2 expression.

## INTRODUCTION

Breast cancer is the most common cancer occurring in women. An estimated 246,660 new cases of breast cancer will be diagnosed in 2016, which are expected to account for 29% of new cancers in women, with an estimated 40,450 deaths [1]. Like other cancers, carcinogenesis of human breast epithelial cells from non-cancerous to pre-malignant is a multiyear, multistep, and multipath disease process involving accumulation of genetic and epigenetic alterations [2]. Invasion and migration are the most ruinous aspects of breast cancer and directly impacts survival probability of patients. However, the underlying mechanisms of these processes in breast cancer remain poorly understood. To achieve more effective treatments of breast cancer and help increase patient survival, it is essential to investigate the mechanisms that drive breast cancer progression.

Nuclear factor erythroid 2-related factor (NRF2) is a transcription factor belonging to the Cap'n'Collar family of leucine-zipper (B-ZIP) proteins. NRF2 integrates cellular stress signals by responding to various oxidative-driven transcriptional events through binding to antioxidant response elements within promoter regions of NRF2 regulated genes [3–6]. Owing to its important role in protecting cells from cytotoxicity associated with reactive oxygen species and electrophilic stressors, NRF2 has been considered a tumor suppresser and its activity can prevents or at least delay carcinogenesis. For instance, the NRF2 activator Sulforaphane has been reported to inhibit the proliferation of human breast cancer cells *in vitro* and suppress the growth and metastasis of orthotopically transplanted breast cancer cells in female athymic mice [7]. However, other studies have shown that NRF2 is aberrantly activated in various breast cancer cells [8–11], and more recent genetic studies of human breast tumors have indicated NRF2 that plays a crucial role in oncogenesis [12, 13].

RhoA belongs to the Ras super family, which is instrumental in regulating cell motility and invasion *in vivo* and *in vitro* [14–16]. RhoA GTPases shuttle between an inactive GDP-bound and an active GTP-bound form and control the assembly of actin stress fibers and limit the extent of the lamellipodium through its downstream effectors mDIA and ROCKs [17–20]. RhoA activity is regulated at the level of protein stability and degradation [21]. Although no constitutively active mutants of Rho GTPases have been detected in human tumors [22–25], a correlation between increased expression of RhoA and poor clinical outcome has been demonstrated in breast cancer by both clinical and experimental data [26–28].

In this study, we examined the role and mechanism of NRF2 in human breast cancer. We demonstrated that NRF2, whose high expression correlates with tumor aggressiveness and poor prognosis, induced RhoA expression by its binding to and silence ERR1 gene and promoted breast cancer cell proliferation and metastasis. Together with other published data, our results showed that inactivation of NRF2 might be helpful for clinic treatments of patients with breast cancer.

## RESULTS

### NRF2 expression is negatively correlated with the outcome of breast cancer patients

**Figure 1:**
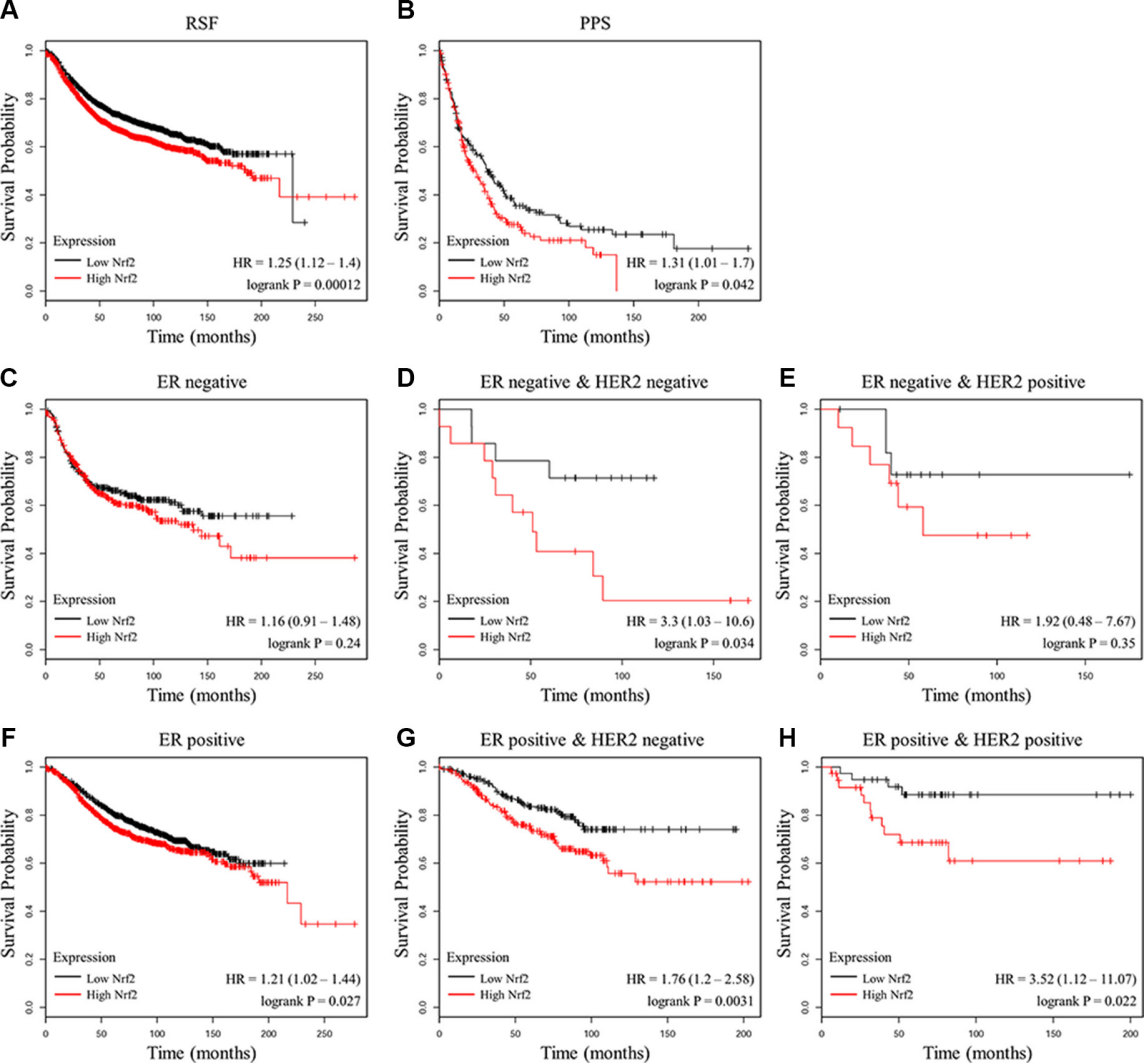
Prognostic significance of NRF2 in breast cancer (**A**, **B**) The effect of NRF2 mRNA expression level on the relapse free survival (A) and post progression survival (B) in 4,142 breast cancer patients was analyzed. The Kaplan-Meier plots were generated by Kaplan-Meier Plotter (http://www.kmplot.com). (**C**–**E**) The effect of NRF2 mRNA expression level on the relapse free survival of ER-negative samples (C), ER-negative and HER2-negative samples (D) or ER-negative and HER2-positive samples (E). (**F**–**H**) The effect of NRF2 mRNA expression level on the relapse free survival of ER-positive samples (F), ER-positive and HER2-negative samples (G) or ER-positive and HER2-positive samples (H).

A previous analysis of 91 patients with estrogen receptor (ER)-positive breast cancer showed that high gene expression level of NRF2 is significantly associated with poor prognosis [29]. To further validate the important role of NRF2 in the outcome of breast cancer patients, we analyzed the relationship between NRF2 mRNA levels and the survival of breast cancer patients in 4142 breast tumor samples using publicly available datasets (kmplot, 2015 version). Kaplan-Meier analyses demonstrated that lower mRNA expression level of NRF2 was correlated with an improvement of relapse free survival (RSF), as well as post progression survival (PPS) of patients (Figure 1A and 1B). These correlations were more significant in ER-negative samples (Figure 1C and 1F). In addition, HER2 expression did not affect these correlations (Figure 1D, 1E, 1G and 1H). These analyses further confirmed NRF2 as a pro-oncogene.

### NRF2 promotes the proliferation and migration of breast cancer cells

**Figure 2:**
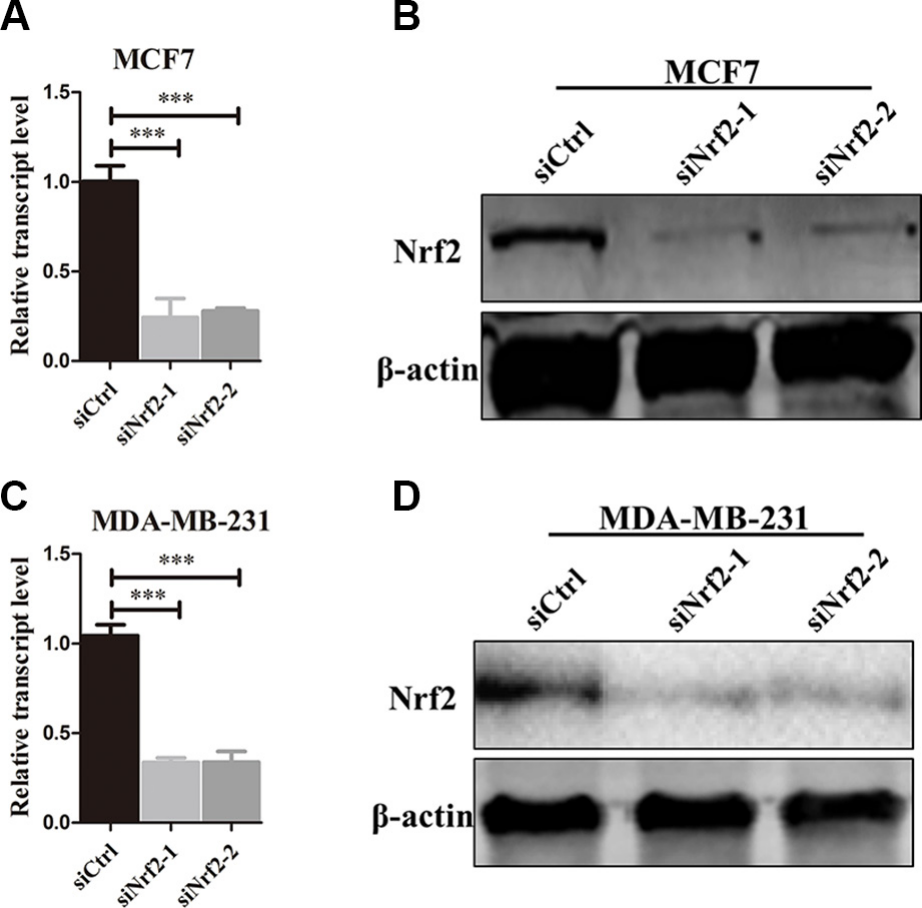
NRF2 is effectively knocked down by siNrf2 (**A**, **B**) NRF2 expression was effectively decreased at both mRNA (A) and protein levels (B) in the MDA-MB-231 cell line. (**C**, **D**) NRF2 expression was effectively decreased at both mRNA (C) and protein levels (D) in the MCF7 cell line. *n* = 3, bar: SD, ***P < 0.005.

**Figure 3:**
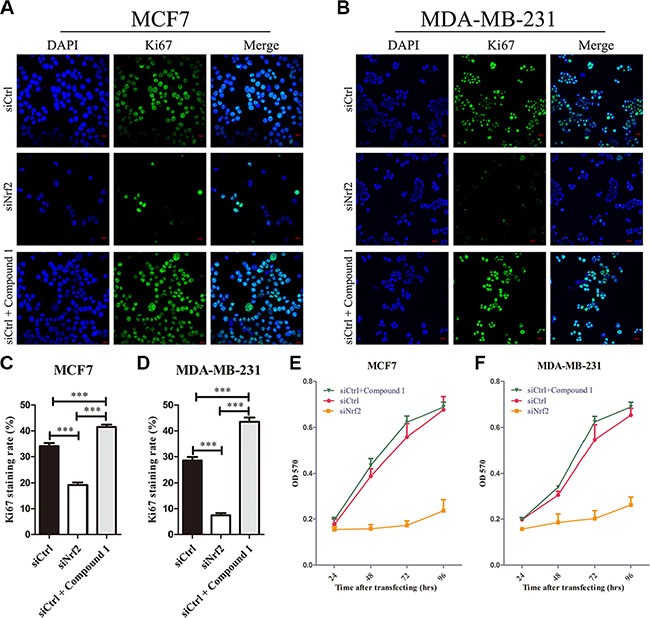
Knockdown of NRF2 inhibits cell proliferation of breast cancer cells Cells were treated with siCtrl, siNrf2 or siCtrl together with Compound 1. (**A**–**D**) Cell proliferation was measured by Ki67 immunostaining. (A, B) Cells were stained with anti-Ki67 antibodies to detect cell proliferation ability (green), and with DAPI, to detect nuclei (blue). *n* = 5. (C, D) Ki67 staining rate was quantified by Image J. (**E**, **F**) Cell growth was measured using thiazolyl blue assay at various time points. *n* = 10, bar: SD, *P < 0.05; **P < 0.01; ***P < 0.005.

To investigate whether NRF2 plays a functional role in breast cancer progression, we first reduced NRF2 expression both at mRNA and protein levels in the MCF7 breast cancer cell line using two small interference RNAs (siNrf2-1 and siNrf2-2) (Figure 2A and 2B). We also confirmed effective knockdown activities in MDA-MB-231 cells (Figure 2C and 2D). We found a remarkable inhibition of cell proliferation in these two breast cancer cell lines as detected by Ki67 immunostaining after NRF2 (Figure 3A–3D) and MTT assay (Figure 3E and 3F). We also found that treatment with Compound 1, an NRF2 small molecule activator we reported previously [30], could enhance cell proliferation of these two breast cancer cells compared to these cells transfected with negative control siRNA (siCtrl) only (Figure 3).

**Figure 4:**
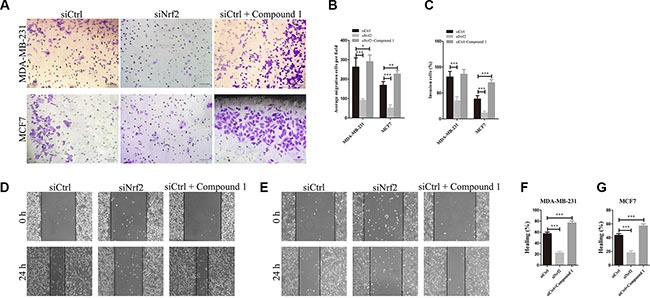
Knockdown of NRF2 inhibits cell metastasis of breast cancer cells Cells were treated with siCtrl, siNrf2 or siCtrl together with Compound 1, respectively. (**A**, **C**) Cell invasion capacity was evaluated by matrigel-coated transwell assay, scale bar: 50 μm. (**B**) cell migration capacity was evaluated by transwell assay. (**D**, **E**) Scratch wound assays of control- (siCtrl) or siNrf2 -transfected and Compound 1-treated MDA-MB-231 cells (D) or MCF7 cells (E). Phase contrast microphotographs are shown. Wound areas are shown as black bars, Scale bar: 100 μm. (**F**, **G**) Quantification is displayed as percentage of maximal migration (area of wound closure in siCtrl-treated cells after 24 h). All data are means of three experiments, bar: SD, *P < 0.05; **P < 0.01; ***P < 0.005.

As tumor metastasis of breast cancer cells is a critical factor that affects RSF and PPS, the role of NRF2 in breast cancer metastasis was evaluated by cell migration and invasion assay. Using transwell assay and wound healing assay, we found that NRF2 silencing significantly decreased cell migration in MDA-MB-231 (Figure 4B, 4D and 4F) and MCF7 cells (Figure 4B, 4E and 4G) (*p* < 0.005). As shown in Figure 4A and 4C, we found that knocking-down of NRF2 significantly reduced the invasion of both MDA-MB-231 and MCF7 cells in the 3D matrigel invasion assay. Surprisingly, decreased cell migration capacity was not as significant in MCF7 cells (Figure 4G) compared with MDA-MB-231 cells (Figure 4F). Notably, MDA-MB-231 is an ER-negative breast cancer cell line exhibited a higher NRF2 expression compared to MCF7, which is an ER-positive cell line. These results consisted with the analysis results above, indicating that NRF2 was able to promote the proliferation and migration of breast cancer.

### NRF2 positively regulates RhoA expression in breast cancer cell

**Figure 5:**
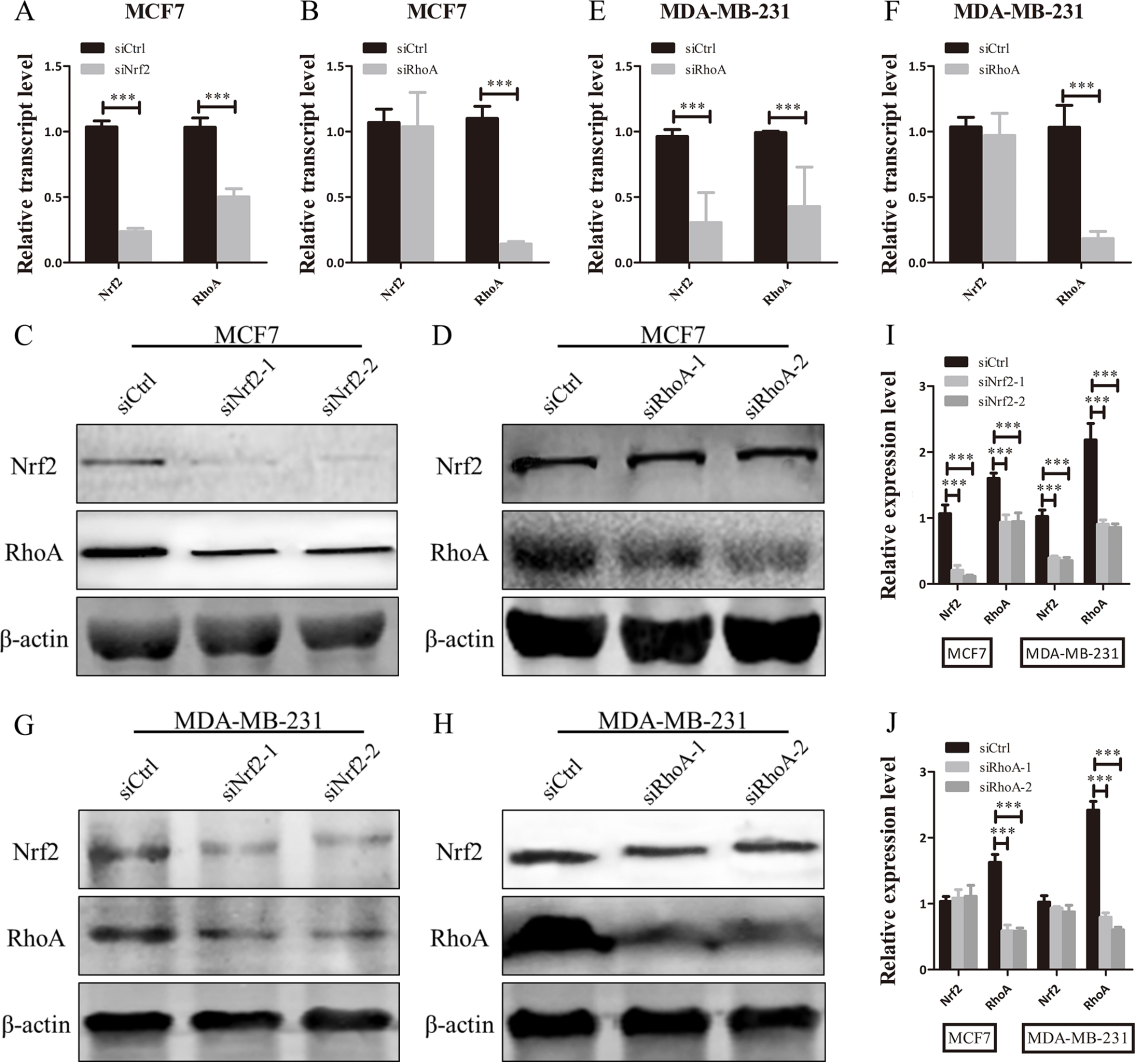
NRF2 promotes the expression of RhoA in breast cancer cells (**A**, **B**, **E**, **F**) qRT-PCR analysis of the mRNA expression levels of NRF2 and RhoA in MDA-MB-231 and MCF7 cells after being transfected with siRNA specifically targeting to the human NRF2 gene or RhoA gene. (**C**, **D**, **G**, **H**) Immunoblotting of NRF2 and RhoA in MDA-MB-231 and MCF7 cells transfected with siNrf2 or siRhoA. *n* = 3. (**I**, **J**) Relative protein expression levels were quantified using ImageJ and normalized to β-actin and then to their corresponding siCtrl -transfected cells. All data are means of three experiments, bar: SD, *P < 0.05; **P < 0.01; ***P < 0.005.

RhoA, as an important small GTPase, is a key factor of cell proliferation and migration in breast cancer [31]. The above results indicated the regulation of cell growth and metastasis in RhoA expression cell lines (Supplementary Figure S3), so we attempted to investigate whether NRF2 could regulate the expression of RhoA. Analysis of TCGA data by cBioPortal showed a positive correlation between NRF2 and RhoA mRNA expression level (Supplementary Figure S1). To further explore the role of NRF2 in the regulation of RhoA expression, we first downregulated NRF2 or RhoA expression in both MDA-MB-231 and MCF7 cells by siRNA transfection and analyzed mRNA and protein levels. Interestingly, we found that RhoA expression was downregulated after suppression of NRF2 expression (Figure 5A, 5C, 5E, 5G and 5I), but NRF2 expression was not as altered after suppression of RhoA expression (Figure 5B, 5D, 5F, 5H and 5J). This positive regulatory role was confirmed in two type breast cancer cell lines with siRNAs targeting different sequences of the NRF2 gene. Taken together, these results suggested that NRF2 positively regulates the expression of RhoA in human breast cancer cells.

### NRF2 deficiency inhibits breast cancer cell growth and metastasis by down-regulation RhoA expression

**Figure 6:**
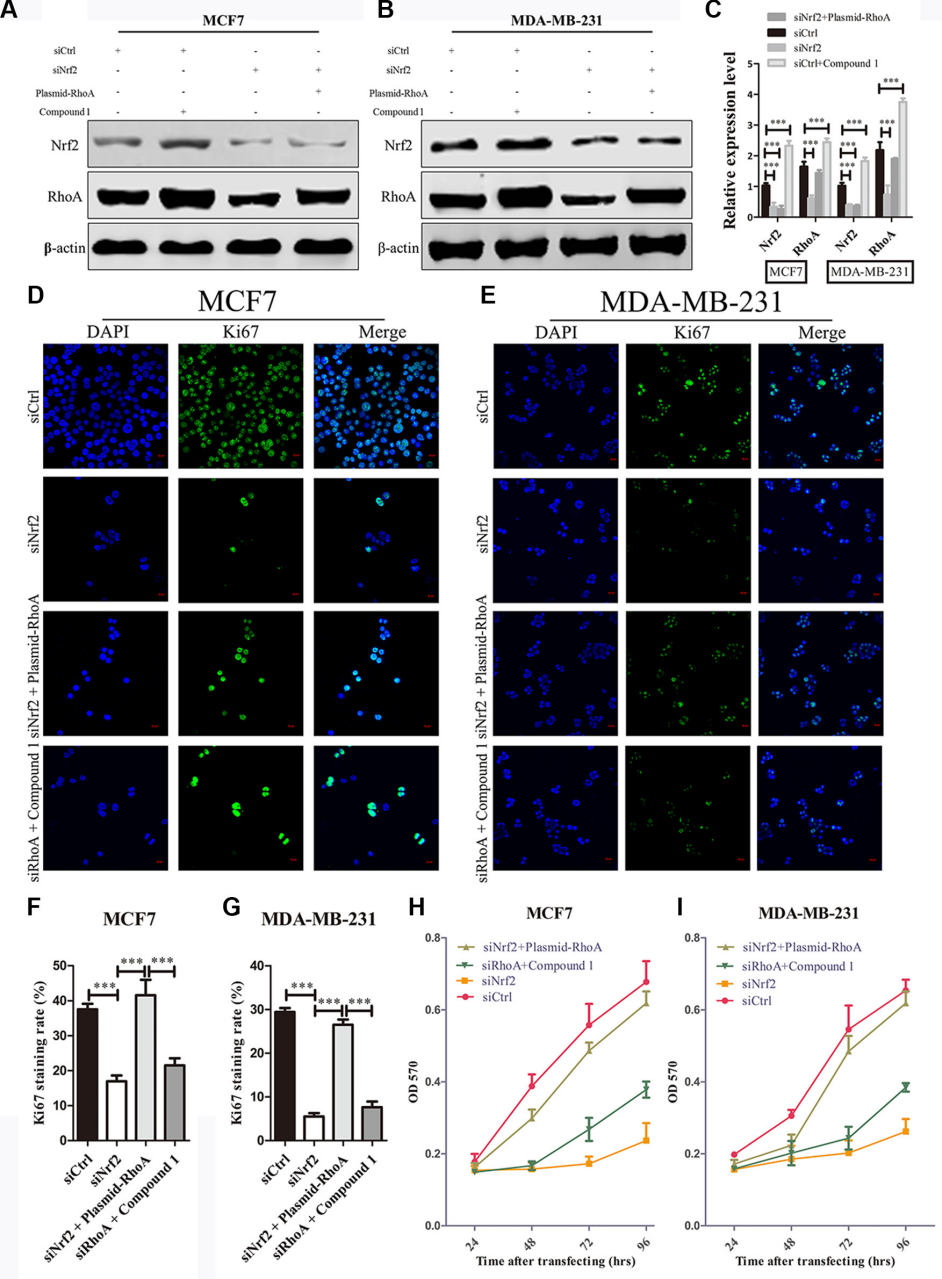
RhoA reverses the effect of NRF2 downregulation on cell proliferation (**A**, **B**) MDA-MB-231 and MCF7 cells were transiently transfected with siNrf2 alone, or simultaneously with RhoA expressing vectors or treated with Compound 1. The protein levels of NRF2 and RhoA were determined by immunoblotting. (**C**) The intensity of each band was quantified using ImageJ and normalized to β-actin and then to their corresponding siCtrl-transfected cells. *n* = 3, bar: SD, ***P < 0.005. (**D**–**G**) Cell proliferation was measured by Ki67 immunostaining. (D, E) Cells were stained with anti-Ki67 antibodies to detect cell proliferation ability (green), and with DAPI, to detect nuclei (blue). *n* = 5. (F, G) Ki67 staining rate was quantified by Image J. (**H**, **I**) Cell growth was determined using thiazolyl blue assay at various time points. Cells were treated with siCtrl, siNrf2 alone, siNrf2 combined with RhoA expressing vectors or siRhoA combined with Compound 1. *n* = 10, bar: SD, *P < 0.05; **P < 0.01; ***P < 0.005.

Aberrantly high expression of RhoA is thought to be a trigger of breast tumor proliferation and metastasis [27]. Therefore, we investigated whether reduced expression of RhoA contributes to the inhibition of breast cancer cell proliferation and metastasis by NRF2 deficiency. We first transfected siNrf2 alone or together with RhoA expressing vectors into MDA-MB-231 and MCF7 cells, and RhoA was either downregulated, or upregulated. And we also found that Compound 1 could increase RhoA expression slightly higher compared with RhoA expressing cells (Figure 6A–6C). The siNrf2-transfected MDA-MB-231 and MCF7 cells with re-expression of RhoA by plasmid transfection exhibited increased cell proliferation compared with cells transfected with NRF2 siRNA alone. Furthermore, Compound 1 could not promote cell proliferation in RhoA silenced MDA-MB-231 and MCF7 cells compared with cells transfected with siCtrl alone, indicating that RhoA is able to reverse the inhibitory effect on cell proliferation by NRF2 downregulation in breast cancer cells (Figure 6D–6J).

**Figure 7:**
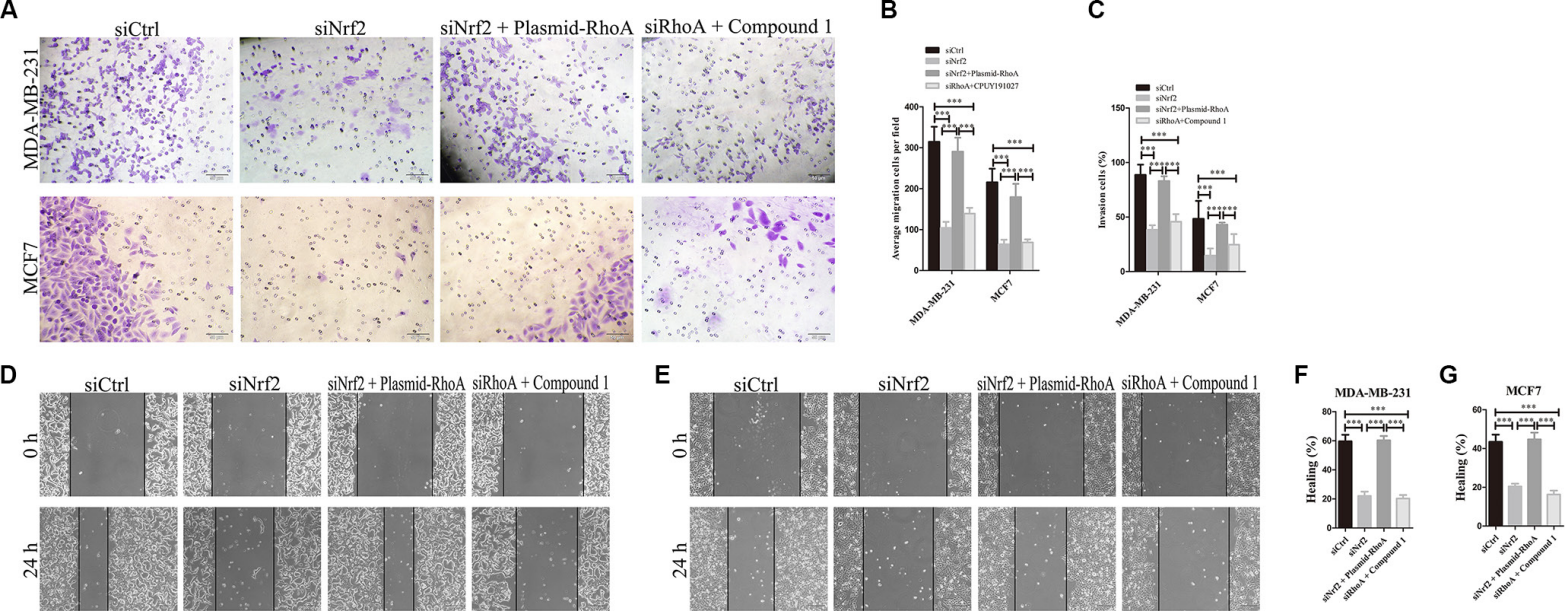
RhoA reverses the effect of NRF2 downregulation on cell metastasis Cells were treated with siCtrl, siNrf2, siNrf2 together with RhoA expressing vectors or siRhoA together with Compound 1. (**A**, **C**) Cell invasion capacity was evaluated by matrigel-coated transwell assays, scale bar: 50 μm. (**B**) Cell migration capacity was evaluated by transwell assay. (**D**, **E**) Scratch wound assays of control- (siCtrl) or other treatment MDA-MB-231 cells (D) or MCF7 cells (E). Phase contrast microphotographs are shown. Wound areas are shown as black bars, scale bar: 100 μm. (**F**, **G**) Quantification is displayed as percentage of maximal migration (area of wound closure in siCtrl-treated cells after 24 h). All data are means of three experiments, bar: SD, *P < 0.05; **P < 0.01; ***P < 0.005.

To investgate the role of RhoA in NRF2-induced tumor metastasis, we also performed cell migration and invasion assays as shown in Figure 7. The migration capacity of siNrf2 and RhoA expressing vectors co-transfected cells was significantly increased compared with siNrf2-transfected cells, but showed little difference with siCtrl-transfected cells. Compound 1 also could not promote breast cancer cell migration in RhoA silenced cells compared with cells transfected with siCtrl alone (Figure 7B, 7D–7G). Similar results were obtained in cell invasion assay (Figure 7A and 7C). Thus, RhoA can reverse the inhibition effect on cell metastasis by NRF2 downregulation in breast cancer cells. These data indicated that RhoA is a downstream effector in the process of NRF2-induced promotion of breast cancer cell proliferation and metastasis.

### NRF2 inhibits downstream signal protein of RhoA

**Figure 8:**
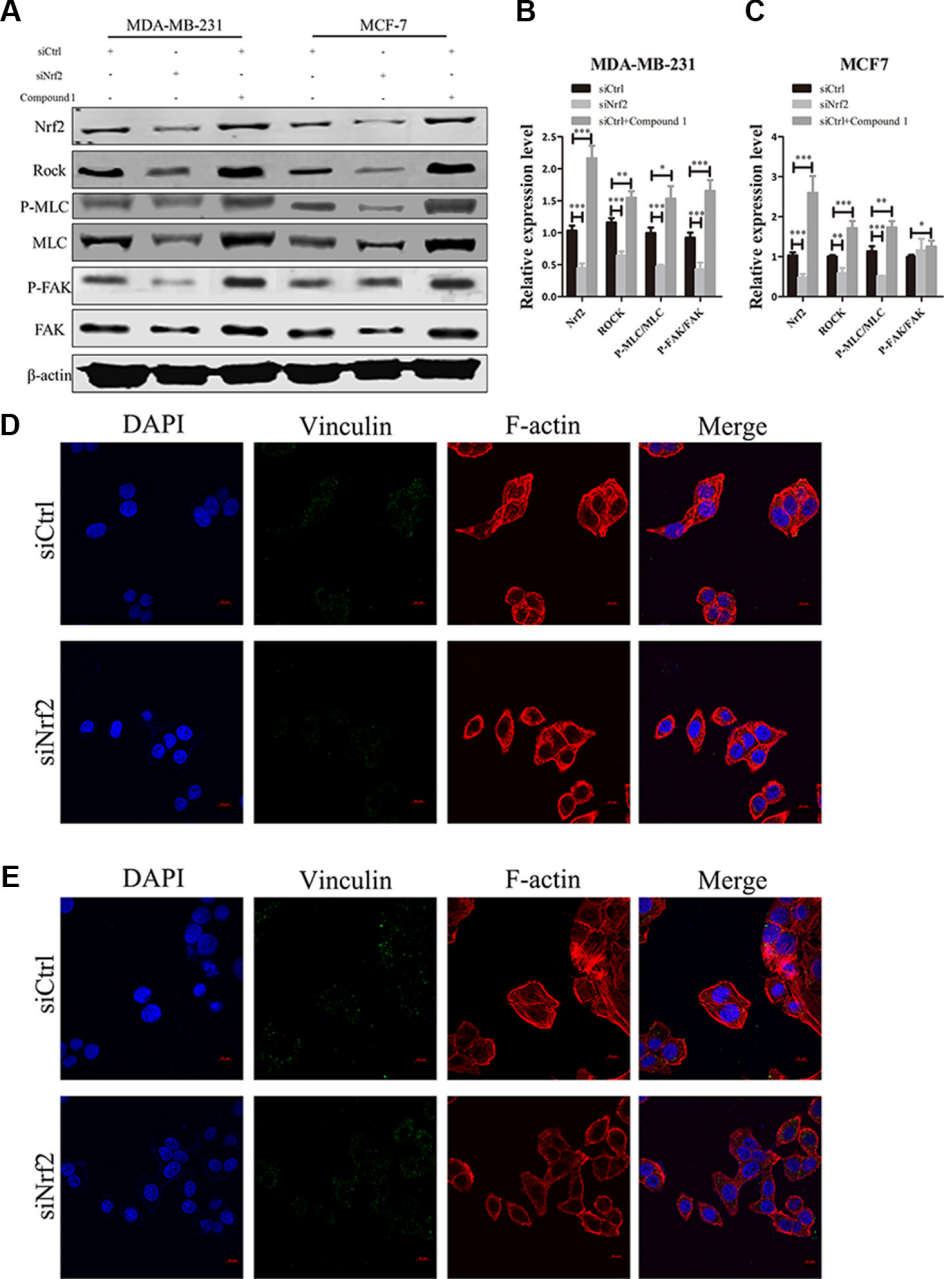
NRF2 effectively promotes downstream signal transduction of RhoA signaling (**A**) Effect of siNrf2-transfection or Compound 1 treatment on ROCK, MLC, phospho-MLC, FAK and phospho-FAK expression. (**B**, **C**) Relative protein expression levels were quantified using ImageJ and normalized to β-actin and then to their corresponding siCtrl -transfected cells. bar: SD, ***P < 0.005. (**D**, **E**) MDA-MB-231 (D) and MCF7 (E) cells were transfected with siCtrl or siNrf2 alone and stained with Rhodamine-phalloidin to detect F-actin stress fibers (red), with anti-vinculin antibodies to detect focal adhesions (green), and with DAPI to detect nuclei (blue). *n* = 5.

We next examined the signaling proteins downstream of RhoA. We found altered protein expression levels of downstream signal protein of RhoA, such as FAK, MLC and ROCK, after NRF2 inhibition or Compound 1 treatment in MDA-MB-231 and MCF7 cells (Figure 8A–8C). Interestingly, phosphor-FAK expression was not significant altered in MCF7 cells after NRF2 silencing. These data suggested the signal transduction of RhoA/ROCK pathway was suppressed after NRF2 downregulation, leading to decreased cell proliferation and metastasis.

We also observed decreased F-actin signal in both siNrf2-transfected MDA-MB-231 and MCF7 cell lines, which demonstrated inhibition of stress fiber formationNRF2 (Figure 8D and 8E). However, the signal of vinculin, a marker of focal adhesion formation, was declined in siNrf2-transfected MDA-MB-231 cells, with almost no changes in siNrf2-transfected MCF7 cells, suggesting that focal adhesion formation was decreased in MDA-MB-231 cells but not in MCF7 cells (Figure 8D). Both stress fiber and focal adhesion formation impacts cell migration and invasion. Taken together, these data demonstrated that NRF2 could activate downstream signal transduction of RhoA, leading to increased formation of stress fiber and focal adhesion, which further influences breast cancer cell metastasis.

### NRF2 inhibits the expression of ERR1 in breast cancer cell

**Figure 9:**
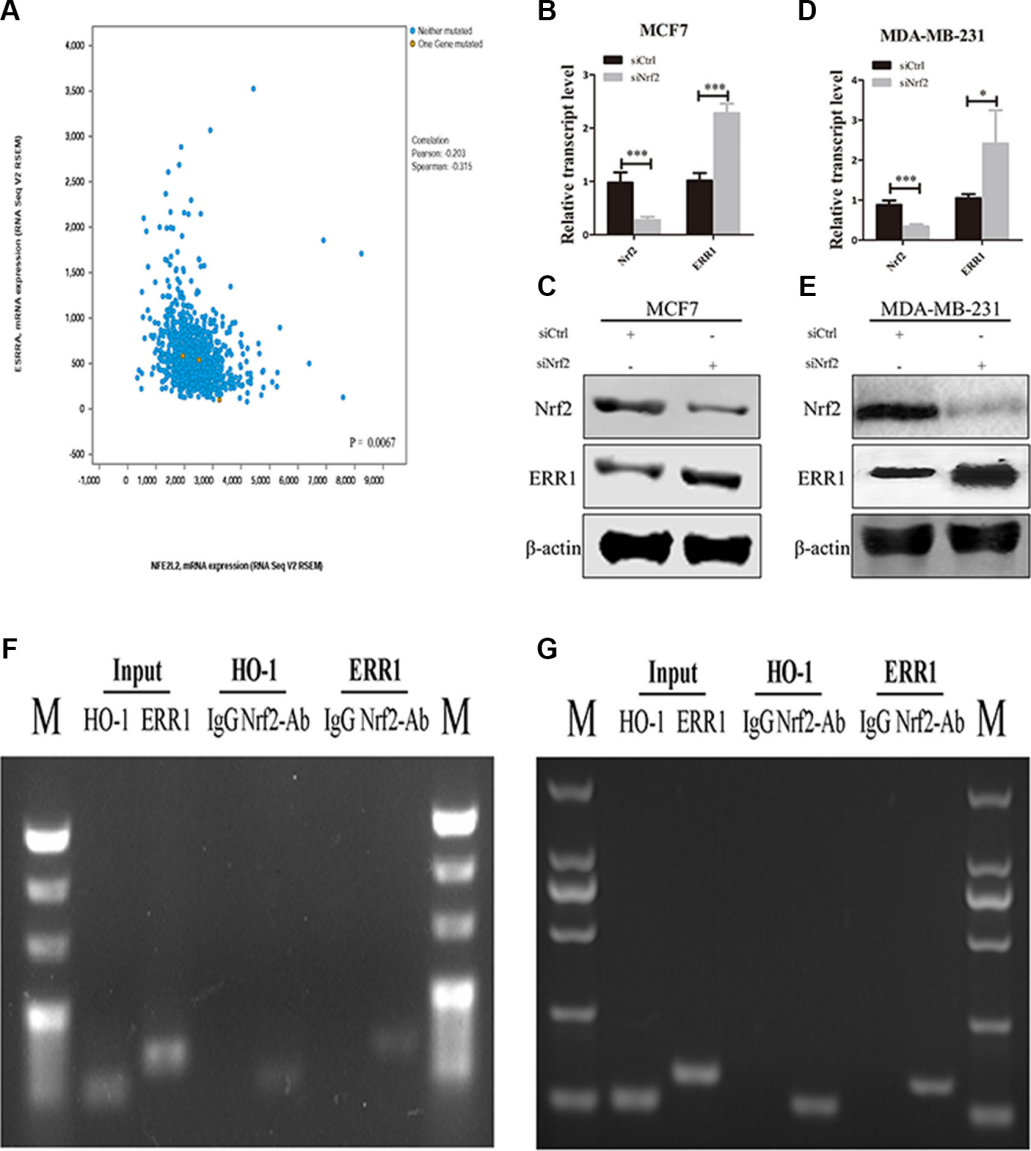
NRF2 negatively regulate the expression of ERR1 (**A**) The relationship between NRF2 and ERR1 mRNA expression was retrieved from TCGA dataset using www.cbioportal.org and the correlation was analyzed by Pearson correlation and Spearman correlation. (**B**, **C**) The expression of ERR1 mRNA and protein levels was measured by qRT-PCR (B) and immunoblotting (C) in MCF7 cells. (**D**, **E**) The expression of ERR1 mRNA and protein levels was measured by qRT-PCR (D) and immunoblotting (E), respectively, in MDA-MB-231 cells. All data are means of three experiments, bar: SD, *P < 0.05; **P < 0.01; ***P < 0.005. (**F**, **G**) Binding of NRF2 to the ERR1 or HO-1 promoter in MCF7 (F) and MDA-MB-231 cells (G). Anti-NRF2- or IgG-immunoprecipitated chromatin was amplified using the indicated primer pairs. Results obtained by real-time PCR are expressed relative to amplified input. *n* = 3. Conventional PCR products from duplicate ChIP were analyzed on agarose gels. Input was diluted 1/100 before PCR. HO-1 gene served as positive control.

One mechanism through which RhoA is downregulated in cancer cells is the ubiquitination of RhoA itself, which is mediated by a protein complex containing CULLIN3 and BTB/POZ domain-containing adapter for cullin3-mediated RhoA degradation (BACURD protein 1 and 2) [32]. Recently, Sailland et al. reported that the nuclear member receptor estrogen-related receptor α (ERR1) could decrease stability of RhoA though regulation of BACURD2 [33]. To investigate whether NRF2 regulates RhoA expression by affecting the expression of these factors, we first examined the mRNA expression pattens of these genes in siCtrl-transfected and siNrf2-transfected cells. We found upregulated mRNA expression levels of ERR1 and BACURD2 in siNrf2-transfected cells compared to controls, while the mRNA expression levels of cul3 and BACURD1 showed little change (Supplementary Figure S2). We analyzed the publicly available TCGA data using cBioPortal. Pearson and Spearman correlation analyses of the RSEM data revealed a significantly negative correlation *(p* < 0.005) between NRF2 and ERR1 mRNA expression levels (Figure 9A). We then knocked down the expression of NRF2 in both MDA-MB-231 (Figure 9D and 9E) and MCF7 cells (Figure 9B and 9C) and determined the change of ERR1 expression at both the mRNA and protein levels. Downregulated NRF2 expression considerably enhanced ERR1 expression, concomitant with the above-described reduced RhoA expression.

To further examine the mechanism underlying the relationship between NRF2 and ERR1, we analyzed the promoter region of ERR1 using JASPAR datasets and first identified potential binding sites for NRF2 (data not shown). Chromatin immunoprecipitation (ChIP) experiments confirmed that NRF2 bound to the ERR1 gene in close vicinity to the putative transcriptional start site in both MCF7 (Figure 9F) and MDA-MB-231 cells (Figure 9G). The NRF2 target gene HO-1 was used as positive control. Together, these data indicated that NRF2 may regulate RhoA expression by inhibiting the expression of ERR1 through its binding to ERR1 promoter region as a silencer, and thus preventing ERR1-mediated degradation of RhoA to further activate a critical signal transduction pathway of RhoA to drive breast cancer progression.

## DISCUSSION

Tumor metastasis in patients with breast cancer indicates poor prognosis and remains the main cause for mortality in these patients. A comprehensive understanding of the cellular factors involved in metastatic dissemination is critical for the development and improvement of novel diagnostic and treatment strategies. As a transcription factor, NRF2 controls the expression of various antioxidant and cytoprotective genes regulating the cellular response to oxidative and electrophilic stress [34]. Owing to its cytoprotective functions, NRF2 has been traditionally studied in the field of chemoprevention [35–38]. However, the negative correlation between NRF2 expression and the outcome of breast cancer patients suggests NRF2 may play an addition role in tumor progression. Evidence has suggested that overexpression or hyperactivation of NRF2 may be involved in tumorigenesis. For instance, NRF2 overexpression has been reported to enhance cell growth of lung cancer by increasing metabolism through the PI3K/Akt pathway [39]. A crosslink between NRF2 signaling and E-cadherin expression demonstrated that NRF2 overexpression could contribute to the invasive potential of malignant cells through deregulation of E-cadherin expression [40, 41]. In this study, for the first time, we report a novel mechanism for the critical role of NRF2 in promoting the proliferation and metastasis of breast cancer. Our data demonstrate that NRF2 binds the ERR1 promoter region as a silencer and inhibits the expression of ERR1, which further stabilizes RhoA protein levels, leading to cytoskeletal changes that underlie cell proliferation and metastasis of breast cancer cells.

Overexpression of RhoA is a common event in breast cancer that promotes tumor cell proliferation and metastasis [42, 43]. Here we demonstrate that recovery of RhoA expression in NRF2-silenced breast cancer cells could rescue NRF2 depletion-induced cell proliferation and metastasis decrease *in vitro*. Combined with our findings that NRF2 modulation more greatly affected MDA-MB-231 cells, which exhibit higher RhoA expression, we inferred that RhoA may be a key factor in the NRF2 deficiency-mediated inhibition of breast cancer cell proliferation and metastasis. Members of the Rho family of GTPases play key roles in cytoskeletal reprogramming by acting as molecular switches that control morphogenesis and movement [15]. RhoA mediates not only polymerization of actin (F-actin formation) to generate stress fibers, which are antiparallel actin filaments that are crosslinked by myosin, but also activation of myosin to trigger contractility [44, 45]. Active (GTP-binding) RhoA binds to Rho-associated coiled-coil-forming kinase (ROCK), resulting in activation of this kinase [46], which promotes the phosphorylation of myosin light chain directly and leads to actin-myosin contraction [47, 48]. For cell moving, the force generated by actin-myosin contractility is used to pull on the extracellular matrix (ECM) at focal adhesions, while ECM stiffness can promote the formation of focal adhesions [49]. We observed a decreased fluorescence signal of polymerized actin after NRF2 silencing in both MDA-MB-231 and MCF-7 cell lines, indicating the down-regulated formation of stress fibers. We also found down-regulated phosphor-MLC protein expression levels in both MDA-MB-231 and MCF-7 cell lines after NRF2 knockdown. Interestingly, we only observed a significant decrease of focal adhesions formation in the MDA-MB-231 cell line but not in MCF-7 cells. These data indicated that NRF2 silencing in both ER-negative and ER-positive breast cancer cells could inhibit RhoA/ROCK pathway signal transduction, while NRF2 deficiency in ER-negative cells could impact formation of both stress fibers and focal adhesions, which implies that NRF2 deficiency could contribute more to inhibition of tumor metastasis potential through more inhibition of the RhoA/ROCK pathway.

In human cancers, RhoA activation and its expression must be tightly regulated for appropriate cellular migration. The most famous factor regulating RhoA expression is a complex containing CULLIN3 and BACURD protein. However, a recent study reported that ERR1, whose high expression correlates with tumor aggressiveness and poor prognosis, decreases the stability and activity of the RhoA protein and promotes cell migration [33]. We observed an increase of ERR1 expression at both mRNA and protein levels and a concomitant decrease of RhoA expression after knockdown of NRF2 in breast cancer cell lines, with no changes in CULLIN3 or BACURD1. We also found that NRF2 bound to the ERR1 gene in close vicinity to the putative transcriptional start site. These results indicate that NRF2 binds the promoter of the ERR1 gene as a silencer and inhibits ERR1 gene expression, which may be the underlying mechanism for the upregulation of RhoA in breast cancer cells. The analyses of publicly available microarray datasets showing an inverse correlation between NRF2 and ERR1 mRNA levels further support our conclusion.

In conclusion, this study for the first time demonstrates the favorable role of NRF2 in the survival of breast cancer patients. Similar to a pro-oncogene, overexpression of NRF2 in breast cancer activates the RhoA gene and its downstream signal proteins, leading to enhanced cell proliferation and metastasis. NRF2 binds the ERR1 promoter region as a silencer, which further increases the expression of RhoA. Therefore, reducing NRF2 expression in breast cancer cells with a malignant phenotype has a potential to develop as a promising strategy to improve the outcome of patients with breast cancer.

## MATERIALS AND METHODS

### Cell culture

Human breast cancer cell lines MCF-7 and MDA-MB-231 were purchased from Type Culture Collection of the Chinese Academy of Sciences (Shanghai, China). MCF-7 cells were grown in DMEM medium supplemented with 10% (v/v) fetal bovine serum (FBS) (Life Technologies, Carlsbad, CA), and maintained in a humidified atmosphere with 5% CO_2_ at 37°C. MDA-MB-231 cells were grown in L-15 medium supplemented with 10% (v/v) fetal bovine serum (FBS), and maintained in none CO_2_ at 37°C. For drug treatment, cells were treated with 20 μM NRF2 activator Compound 1 for 24 h.

### Plasmids, siRNA and transfection

NRF2 siRNA, RhoA siRNA and a scramble non-targeting siRNA (siCtrl) were purchased from Biomics Biotech (Biomics Biotechnologies Co., Ltd, Nan Tong, China). The siRNA sequences are as follows: siNrf2-#1: 5′-GAGACUACCAUGGUUCCAA-3′, siNrf2-#2: 5′-GUG AGAACACACCAGAGAA-3′, siRhoA-#1: 5′-CAGAUA CCGAUGUUAUACU-3′, siRhoA-#2: 5′-AAGGCAGAG AUAUGGCAAA-3′, and siCtrl: 5′-UUCUCCGAACGU GUCACGU-3′. The pcDNA3.1-RhoA plasmid was constructed by inserting the corresponding cDNA fragments from pGEX-2T-RhoA (Addgene plasmid #12202, ref:[50]) into the pcDNA3.1 vector. The plasmids and siRNA were transfected into cells by using Lipofectamine 2000 reagents (Life Technologies, Carlsbad, CA) according to the manufacturer's instructions.

### RNA extraction and quantitative reverse transcription PCR (qRT-PCR)

Total RNA was extracted using Trizol reagent (Life Technologies, Carlsbad, CA), and the first strand cDNA was generated by the Reverse Transcription System (Takara, Japan, Cat No.RR047A) in a 20 μl reaction containing 1 μg of total RNA. A 1 μl aliquot of cDNA was amplified by the SYBR Green PCR Master Mix (Takara, Japan, Cat No.RR820A) in each 20 μl reaction. PCR reactions were run on the ABI StepOne plus Real-Time PCR system with the following primers: NRF2, forward, 5′-TGACAATGAGGTTTCTTCGGC-3′, reverse, 5′-TGTCCTGTTGCATACCGTCT-3′; RhoA, forward, 5′- GACTCGGATTCGTTGCCTGA-3′, reverse, 5′-GCCAA CTCTACCTGCTTTCCA-3′; ERR1, forward, 5′-CTGGTG GTTGAGCCTGAGAAGC-3′, reverse, 5′-CAGACAGCG ACAGCGATGAGAA-3′; cul3, forward, 5′-AGTCCCTC GCCTGTGGTAAACC- 3′, reverse, 5′-CCTCTCTGGGT CGGATTCACCT-3′; Bacurd1, forward, 5′-CCGCTGACC CCGAACAG-3′, reverse, 5′-CCGGCAGTGGCACAGAC C-3′; Bacurd2, forward, 5′-CTCAGAACCGGCAAGAAA TC-3′, reverse, 5′-ATGTTGCACACAGGCTGGTA-3′; GAPDH, forward, 5′-AGAAGGCTGGGGCTCATTTG-3′, reverse, 5′-AGGGGCCATCCACAGTCTTC-3′. The relative expression values of NRF2, RhoA and ERR1 were calculated and normalized to GAPDH in each sample. The experiments were performed in triplicates.

### Western blot analysis

Whole cells were lysed with RIPA lysis buffer (Beyotime, China, Cat No.P0013B). After protein quantification with the BCA Protein Assay Kit (Thermo Fisher Scientific, CA, Cat No.23225), equal amounts of proteins were separated on SDS-PAGE, and transferred to a PVDF membrane. Antibodies against the following proteins were used: Rabbit-anti-NRF2, rabbit-anti-MLC2, rabbit-anti-phospho-MLC2 (Thr18/Ser19), rabbit-anti-FAK, rabbit-anti-phospho-FAK (Tyr397), and rabbit-anti-ERR1, all purchased from Cell Signaling (Cat No. 12721, 3672, 3674, 3285, 8556 and 13826, respectively). Mouse-anti-RhoA was purchased from Cytoskeleton (Cat No. ARH03, CA), and mouse-anti-actin was purchased from ProteinTech (Cat No. 60008-1-Ig, CA).

### Cell proliferation assay

Cells were seeded in 96-well plates at an initial density of 5000 cells/well, and siRNA transfection was performed on the second day. On the following days, 10 μl Thiazolyl blue (MTT)was added to each well and cells were incubated at 37°C for 4 hs. The medium was removed carefully and 150 μl DMSO was added followed by gentle shaking. Optical density of the released color was read at 570 nm.

### Cell migration assay

MCF7 and MDA-MB-231 cells were resuspended in 500 μl DMEM and L15 medium containing 1% (v/v) FBS, and seeded in the upper transwell chamber (8 μm pore size, Corning, Cat No. 354578). Cells were allowed to migrate toward lower chamber containing 600 μl DMEM or L-15 medium with 10% (v/v) FBS for 22 h. The migrant cells attached to the lower chamber were stained with 0.1% crystal violet and quantified. Each assay was performed three times in triplicates.

### Wound healing assay

Cells were cultured in 6-well plates until full confluence. The cell monolayer was carefully scratched using a 200 μl sterile pipette tip and washed twice with fresh medium. Cells were cultured in the presence of 5 μg/ml of mitomycin C to inhibit cell proliferation. The wound edges were photographed under an inverted-phase microscope after 24 h, and measured.

### Cell invasion assay

Cells were seeded in the top of Matrigel invasion chambers (8 μm pore size, Corning, Cat No. 354480) and allowed to migrate toward the lower chamber for 22 h. Matrigel was removed using cotton buds and the migrant cells were fixed for 30 min with 4% (wt/vol) formaldehyde, colored with 0.1% crystal violet, and microphotographed. Each assay was performed three times in triplicates.

### Chromatin immunoprecipitation (ChIP)

ChIP experiments were performed using the ChIP Assay Kit (Beyotime, China, Cat No.P2078) according to the manufacturer's protocol. Cells were incubated for 10 min in PBS containing 1% formaldehyde and for 5 min in 0.125 M glycine. After centrifugation, cell pellets were resuspended in lysis buffer (1% SDS, 50 mM Tris·HCl pH 8, 10 mM EDTA). Sonication was performed with Bioruptor (Diagenode). Lysates were precleared with 30 μL of protein A-agarose, incubated with 10 μg of anti-NRF2 antibody (Abcam, CA, Cat No. ab62352) or rabbit IgG(Abcam, CA, Cat No. ab172730) overnight at 4°C on rotation, followed by incubation with 30 μL of protein A-agarose for 2 h. After washing the immune complexes were eluted from beads in a buffer containing 1% SDS and 0.1 M NaHCO_3_. Cross-linking was reversed overnight at 65°C, and DNA fragments were purified using the QIAquick column (Qiagen, Germany, Cat No. 28106). Quantitative PCRs were performed using 2 μL of DNA in triplicate.

### Primers for ChIP experiments were as follows

ERR1 proximal: AGGAGAATCGCTTGAACC; ERR1 distal: CGTGCAATATTTGGGACAT.

HO-1 proximal: TCATCCTGTTGCTTGACTAA; HO-1 distal: GTTGTTCTGGTCCTCTAGG.

### Immunofluorescence

MCF7 and MDA-MB-231 cells were washed twice with cold PBS after siRNA or plasmid transfection, and fixed with 4% (w/v) formaldehyde at room temperature for 30 min. The fixed cells were washed three times with PBS containing 0.1% Triton X-100, followed by three washes with PBS. The fixed cells were blocked with 5% (w/v) BSA, and stained with an appropriate primary antibody (anti-Vinculin, 1:200 Abcam, CA, Cat No. ab73412; anti-Ki67, 1:50, Abcam, CA, Cat No. ab16667), followed by Fluor488-conjugated secondary antibody (Invitrogen, CA, Cat No. A21202). For stress fiber formation assays, the cells were stained with Rhodamine Phalloidin for 30min (Cytoskeleton, Cat No. PHDR1). The coverslips were mounted in Vectashield mounting medium with DAPI. The fluorescence images were obtained with an Olympus fluorescence microscope.

### Statistical analysis

Results are presented as the mean ± SD for at least three independent experiments for each group. Chi-squared exact test and Spearman correlation were applied to analyze the association between the expression of NRF2 and RhoA. Statistic differences were determined using ANOVA or two sample *t*-tests for independent samples. Linear mixed effects models were used for analysis to take account of correlations among correlated observations, such as the cell growth measured over time in cell culture. *P* values less than 0.05 were defined as statistically significant after adjustment for multiple comparisons using Holm's procedure.

## SUPPLEMENTARY MATERIALS FIGURES


