# Supplementary material for: NRF2 promotes breast cancer cell proliferation and metastasis by increasing RhoA/ROCK pathway signal transduction
Source: Oncotarget. 2016 Oct 4;7(45):73593–606. doi: 10.18632/oncotarget.12435 (PMC5342001; doi:10.18632/oncotarget.12435)
Supplement: Supplementary file 1 [file oncotarget-07-73593-s001.pdf]

NRF2 promotes breast cancer cell proliferation and metastasis by increasing RhoA/ROCK pathway signal transduction

Supplementary Materials

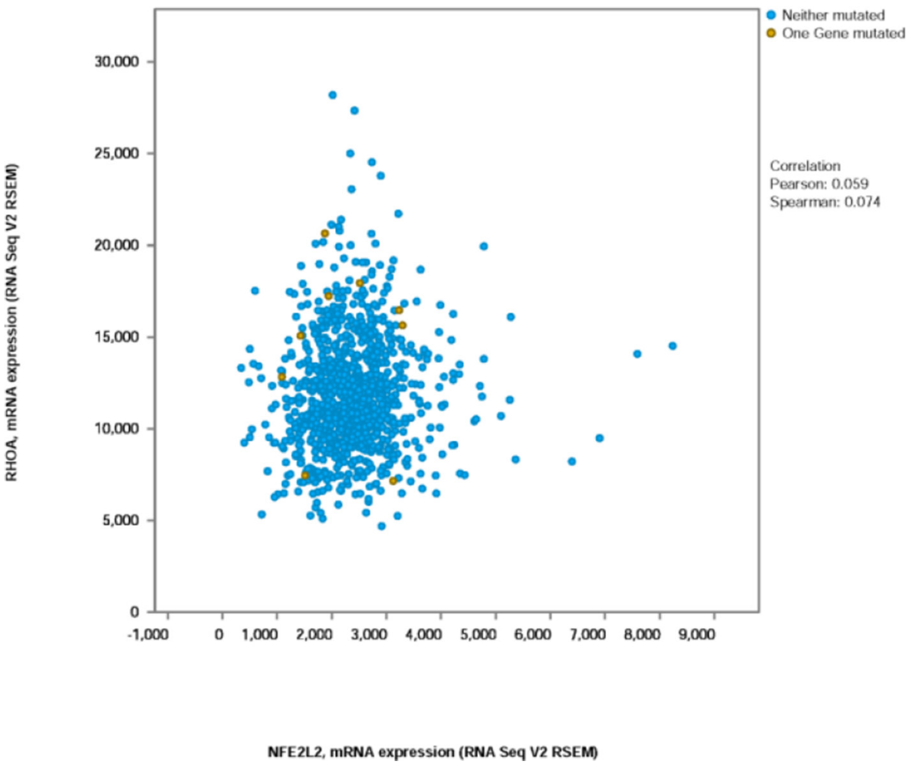

Supplementary Figure S1: The relationship between Nrf2 and RhoA mRNA level in human breast cancer. The Nrf2 and RhoA mRNA expression data of human breast cancer were retrieved from TCGA dataset. The patients were distinguished based on their Nrf2 mRNA expression levels, and the RhoA mRNA level detected by RNA seq V2 RSEM was plotted.

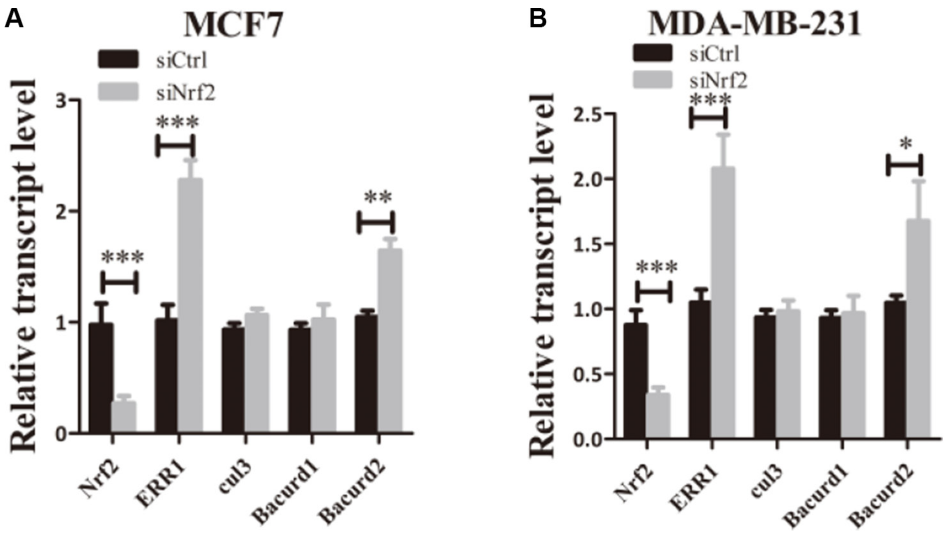

Supplementary Figure S2: Nrf2 inhibits the expression of ERR1 in breast cancer cells. (**A**) qRT-PCR was conducted to determine the mRNA expression levels of Nrf2, ERR1, cul3, Bacurd1 and Bacurd2 in MCF7 cells after being transfected with siRNA specifically targeting to the human Nrf2 gene. (**B**) qRT-PCR was conducted to determine the mRNA expression levels of Nrf2, ERR1, cul3, Bacurd1 and Bacurd2 in MDA-MB-231 cells after being transfected with siRNA specifically targeting to the human Nrf2 gene.

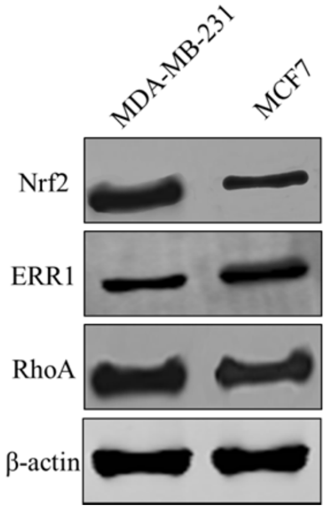

Supplementary Figure S3: The expression level of Nrf2, ERR1 and RhoA in various breast cancer cell lines.
